# Supplementary material for: Metformin and Androgen Receptor-Axis-Targeted (ARAT) Agents Induce Two PARP-1-Dependent Cell Death Pathways in Androgen-Sensitive Human Prostate Cancer Cells
Source: Cancers (Basel). 2021 Feb 5;13(4):633. doi: 10.3390/cancers13040633 (PMC7914929; doi:10.3390/cancers13040633)
Supplement: Supplementary file 1 [file cancers-13-00633-s001.pdf]

Supplementary Materials: Metformin and Androgen Receptor-Axis -Targeted (ARAT) Agents Induce Two PARP-1-dependent Cell Death Pathways in Androgen-sensitive Human Prostate Cancer Cells

Yi Xie, Linbo Wang[1], Mohammad A. Khan, Anne W. Hamburger, Wei Guang, Antonino Passaniti, Kashif Munir, Douglas D. Ross, Michael Dean and Arif Hussain

A.

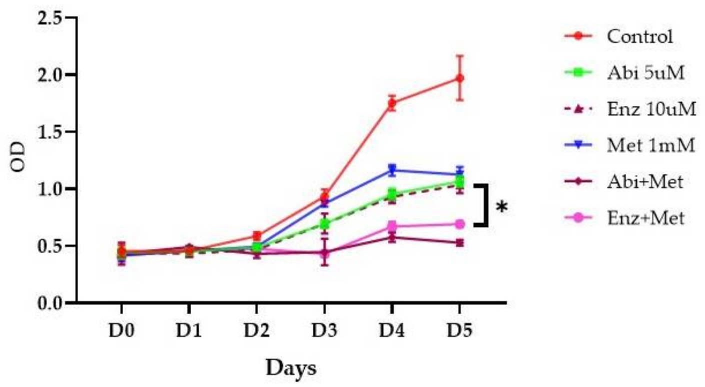

B.

	Control	Abi 5uM	Enz 10uM	Met 1mM	Abi+Met	Enz+Met
PDT(hr)	42±1.1	64.6±1.3	67.1±0.6	67.8±1.6	162±9.1	111.7±3.9

Figure S1. (**A**). Cell growth assays. LNCaP cells were plated in 96-well plates at a density of 3000 cells/well. After two days cells were treated with DMSO (control), Abi, Enz, Met, Abi + Met, Enz + Met, with each treatment done in quadruplicates for the indicated time points. MTT was added and after dissolving the formazan crystals optical density (OD) read at 570 nm. (**B**). The population doubling times (PDT) under basal conditions and after different treatments were determined by plotting the OD readings at the 'exponential growth phase' with respect to time (in hours) using the "Doubling Time" algorithm developed by Roth (Roth V. (2006). Doubling Time Calculator; https://www.doubling-time.com/compute_more.php)

Combination index (CI) for Abiraterone and Metformin

Cell line and Fractions affected (fa)	CI	Synergy Level	*Description
LNCaP			
0.50	0.93827	±	Nearly additive
0.75	0.82288	++	Moderate synergism
0.90	0.72206	++	Moderate synergism

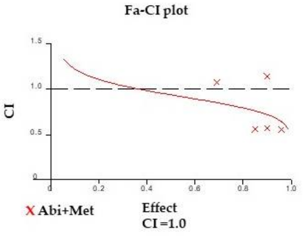

Combination index (CI) for Enzalutamide and Metformin

Cell lines and Fractions affected (fa)	CI	Synergy Level	Description
LNCaP			
0.50	1.09557	±	Nearly additive
0.75	0.84923	++	Moderate synergism
0.90	0.75957	++	Moderate synergism

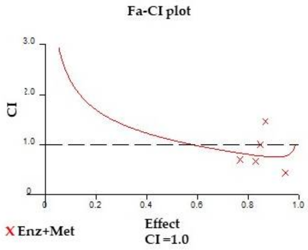

Figure S2. IC50 values and Combination Index for metformin, Abi and Enz.

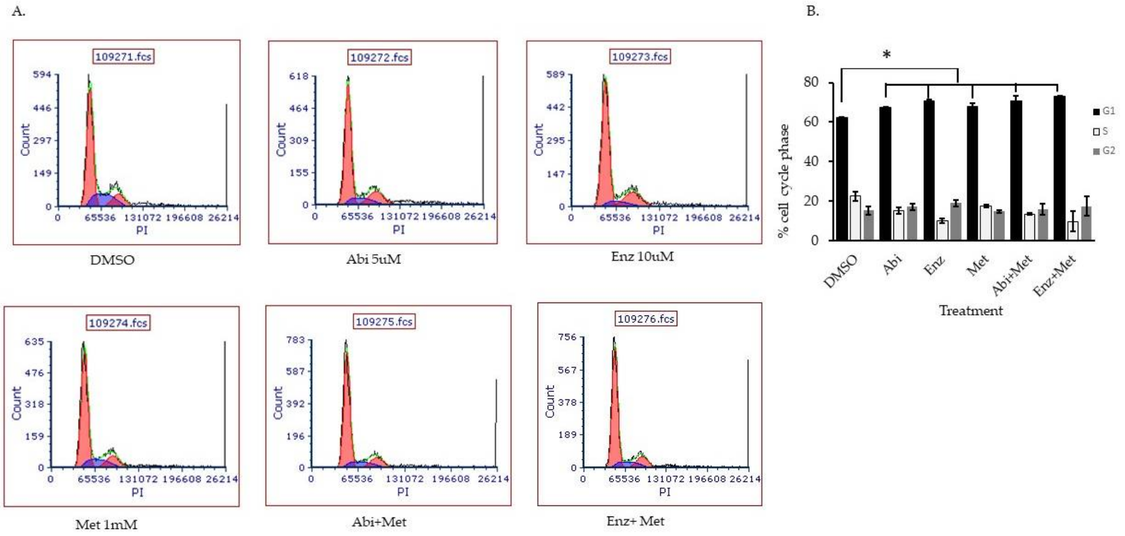

Figure S3. (A). Cell cycle analysis. Cells were trypsinized, washed with PBS twice and fixed in ice-cold 70% ethanol for 60 min, centrifuged and resuspended in 50 µg/mL propidium iodide (PI). A BD FACSCanto-II Flow Cytometer was used to detect the different cell cycle phases, and data analyzed with FCS Express 7 and plotted using GraphPad Prism (v.8) software. (**B**). Data summary of A. Results are the mean of two independent experiments, each consisting of three replications, * ($p < 0.05$), student's *t*-test.

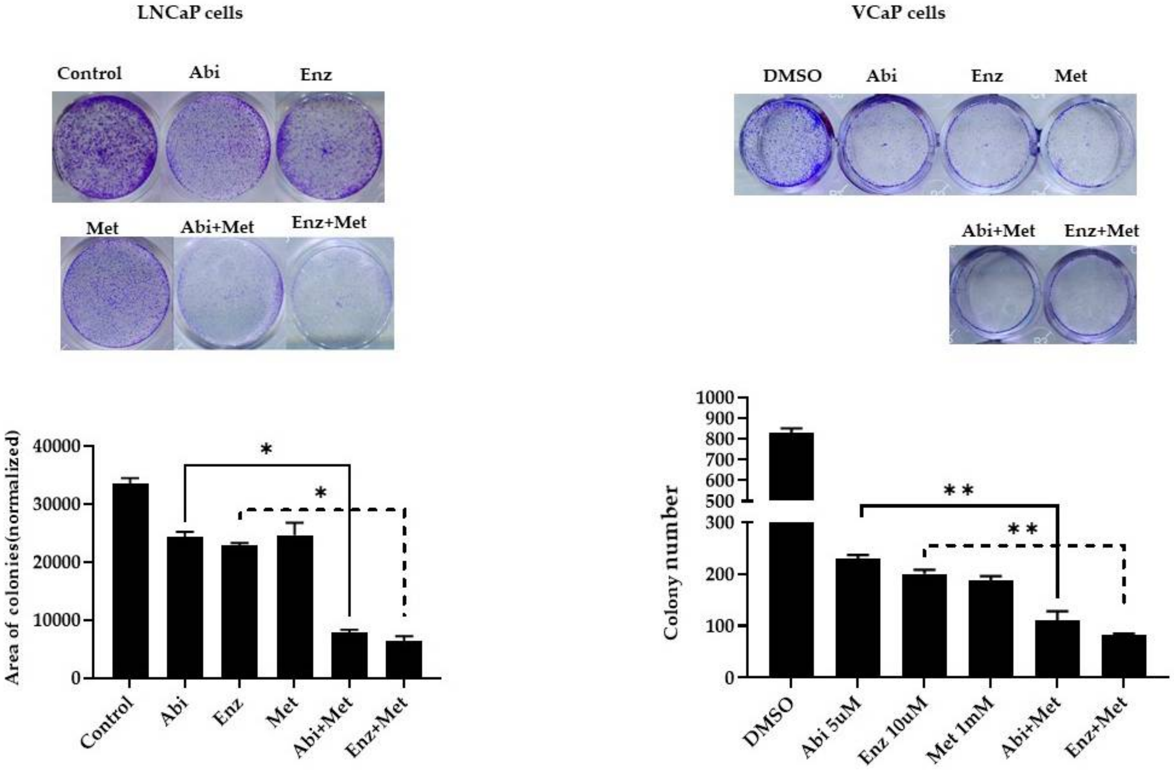

Figure S4. 2-D clonogenic assay for LNCaP (left) and VCaP cells (right). The plated cells were treated with abiraterone (Abi) 5 μM, enzalutamide (Enz) 10 μM, and metformin (Met) 1 mM for over a week, then fixed with ethanol and stained with 0.5% crystal violet. ImageJ (1.48v) software was used to determine the area covered by the colonies and graphed with Excel program. Results are the mean of two independent experiments, each consisting of three replications, * ($p < 0.05$), student's *t*-test.

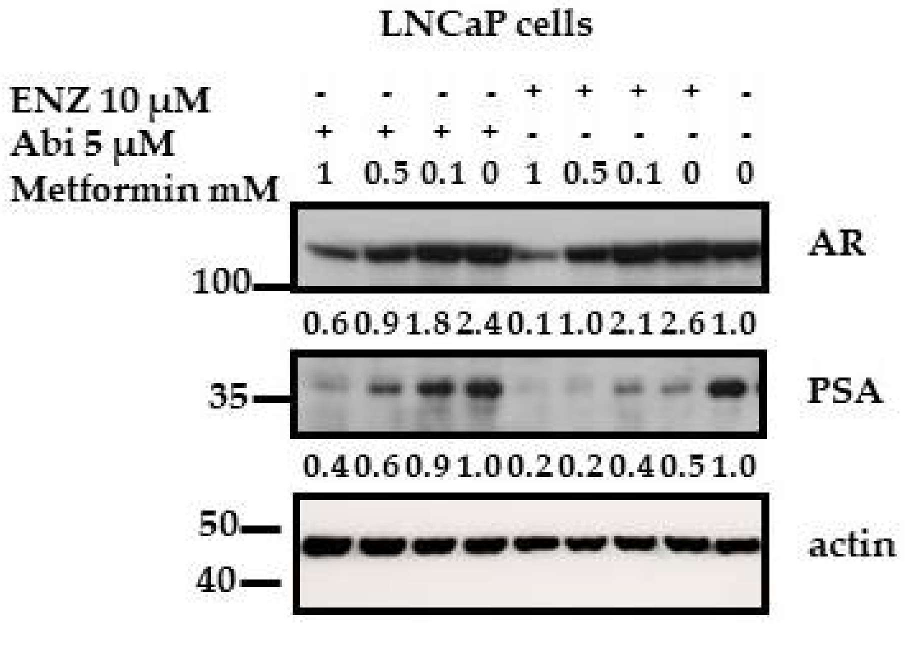

Figure S5. Western blot. AR and PSA expression.

Original western blots for Figure 1E&1G.

Fig 1E. Western blot. Effect of ARATs on AR and PSA expression in PC cells

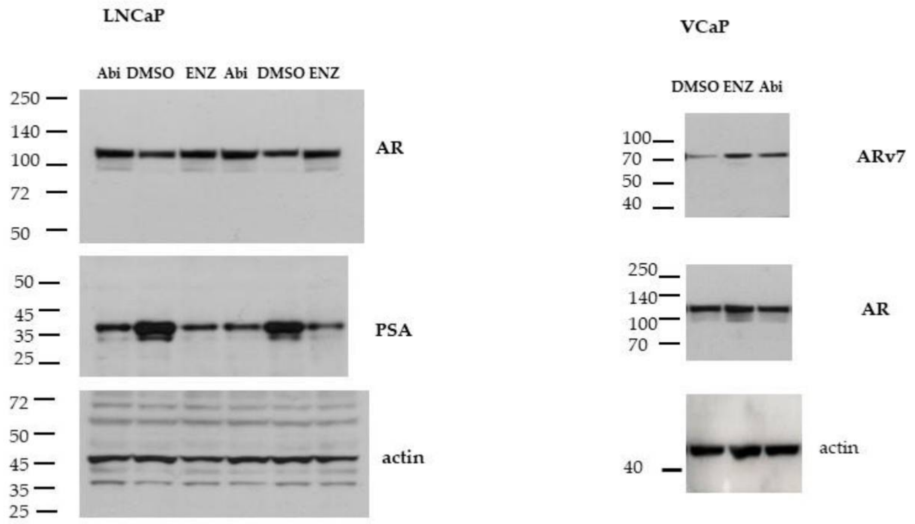

Fig 1G. Western blot. cPARP-1 in ARAT-treated PC cells

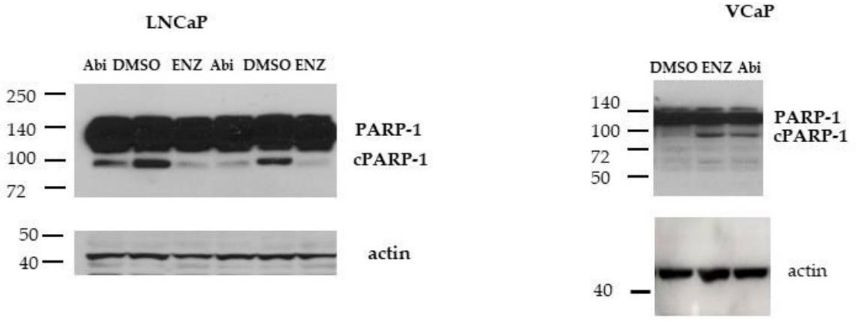

Figure S6. Original western blots for Figure 1E,G.

Original western blots for Figure 2E, 2F&2H.

Fig 2E. Western blot. AR, PSA expression. Dose- and time-course of metformin treatment in LNCaP cells

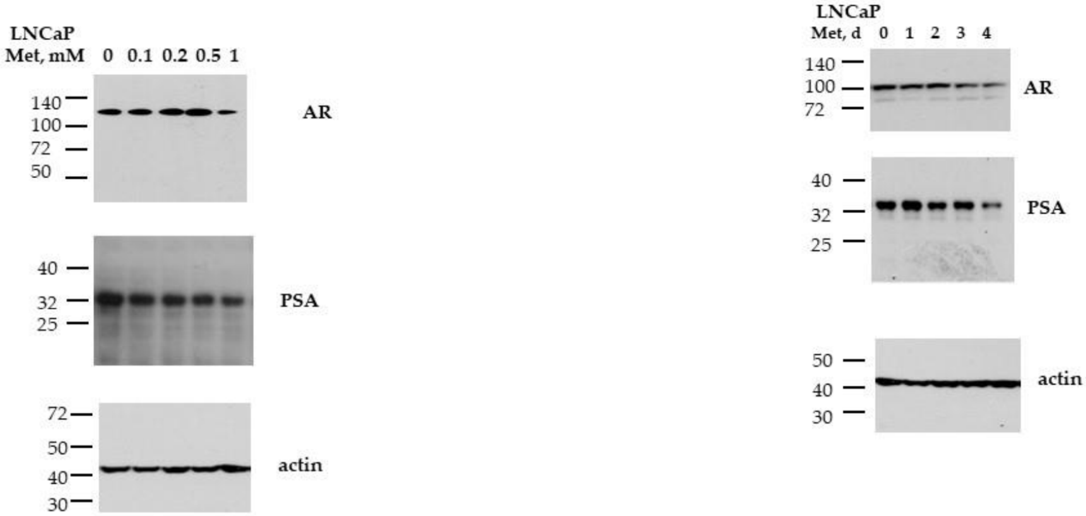

Fig 2F. Western blot. AR, AR-v7 expression. Dose- and time-course of metformin treatment in VCaP cells

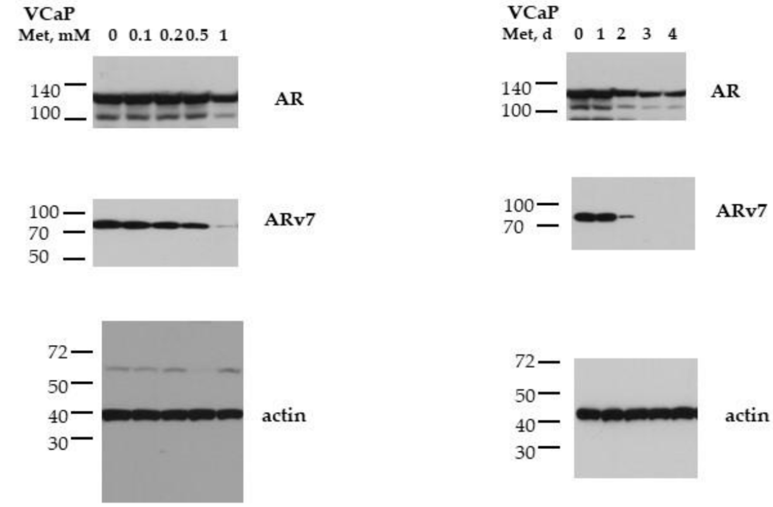

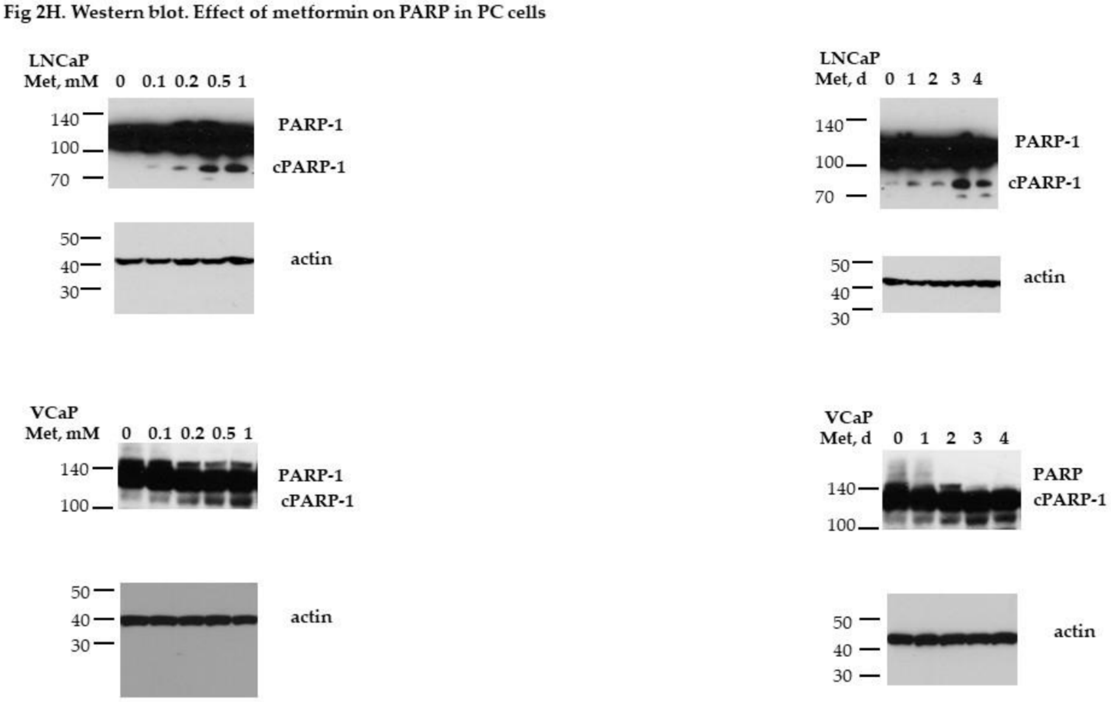

Figure S7. Original western blots for Figure 2E,F,H.

Original western blots for Figure 3C, 3E, 3G, 3H&3L.

Fig 3C. Western blot. AR, AR-v7 expression in metformin plus ARAT treated LNCaP cells

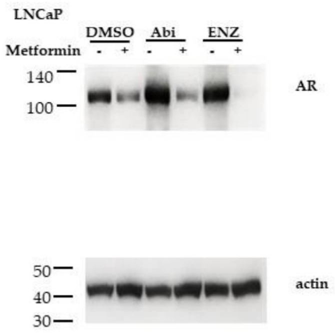

Fig 3D. Western blot. AR, AR-v7 expression ir metformin plus ARAT treated VCaP cells

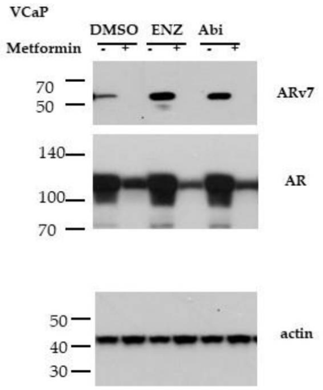

Fig 3E. Western Blot

LNCaP

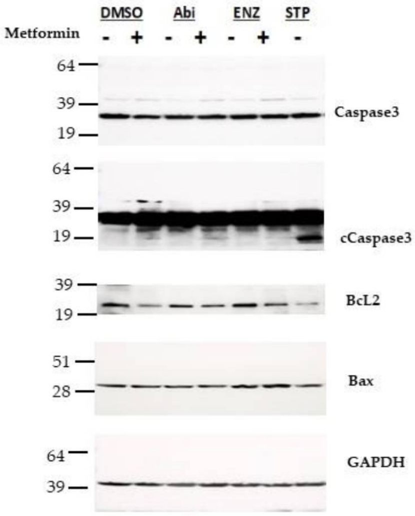

VCaP

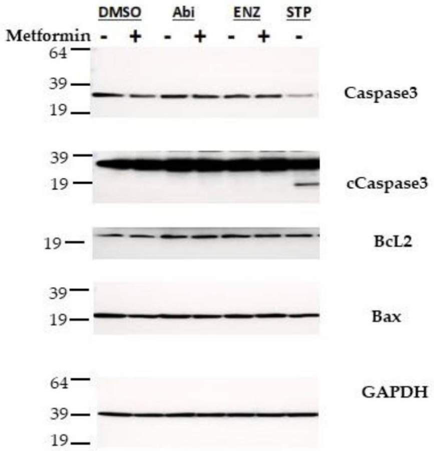

Fig 3G. Western blot

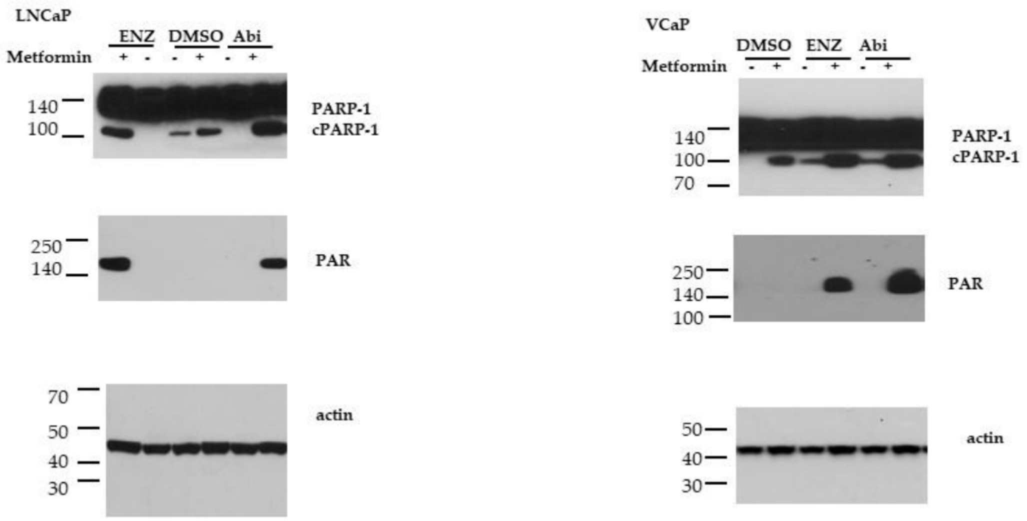

Fig 3H. Western blot. LNCaP cells. PARP-1 silencing

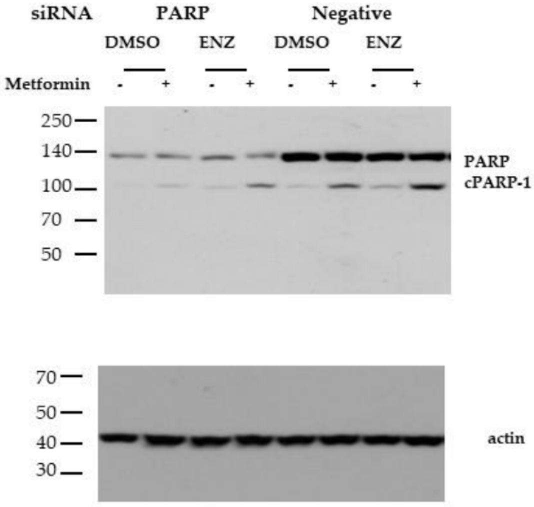

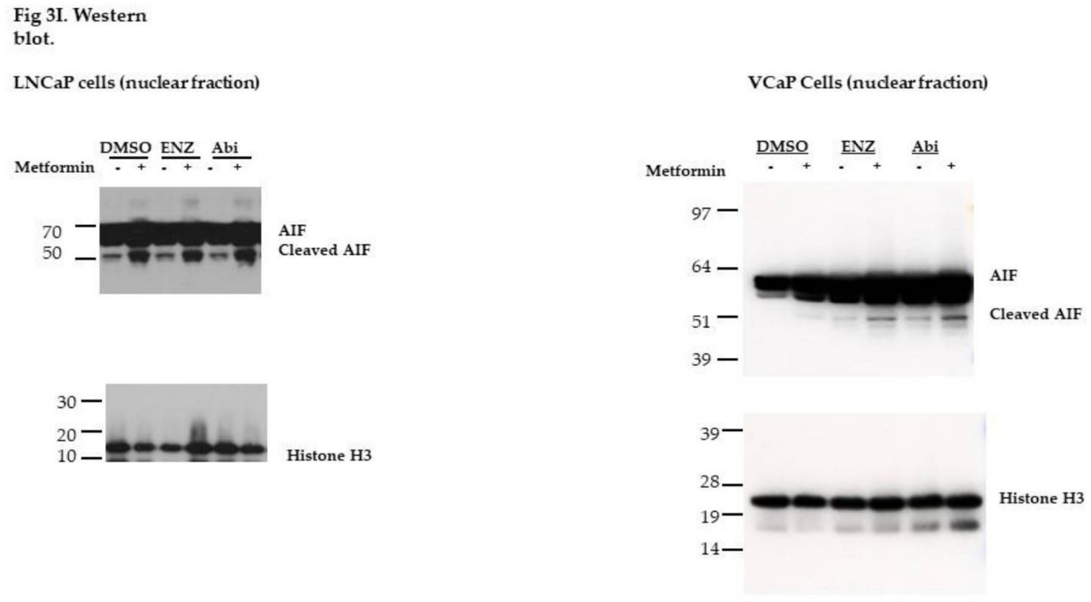

Figure S8. Original western blots for Figure 3C,E,G,H,L.

Original western blots for Figure 4C, 4D&4E.

Fig 4C. Western blot. Effect of lysosome inhibitor chloroquine.

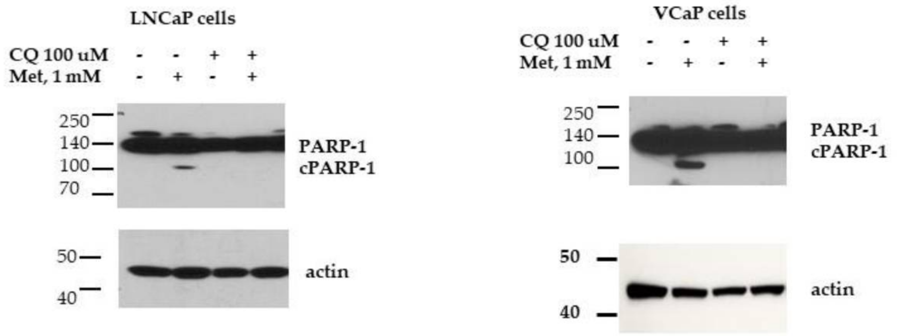

Fig 4D. Western blot. Effect of cysteine protease inhibitor, E-64d

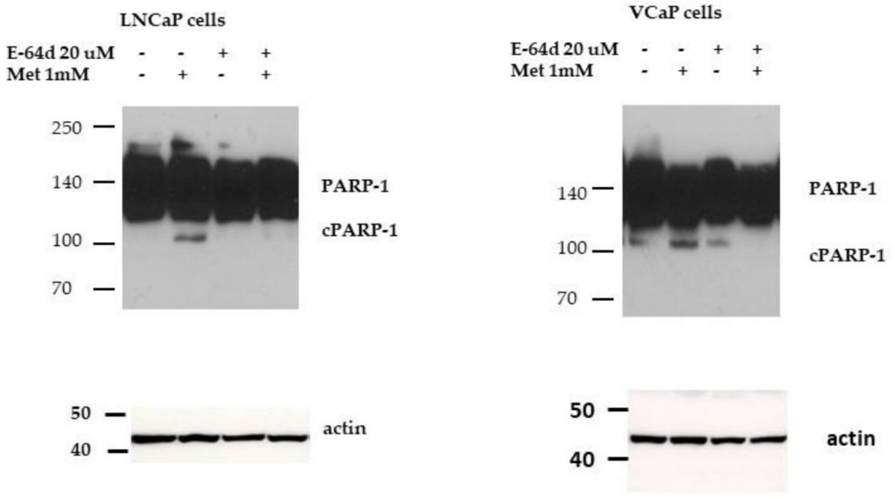

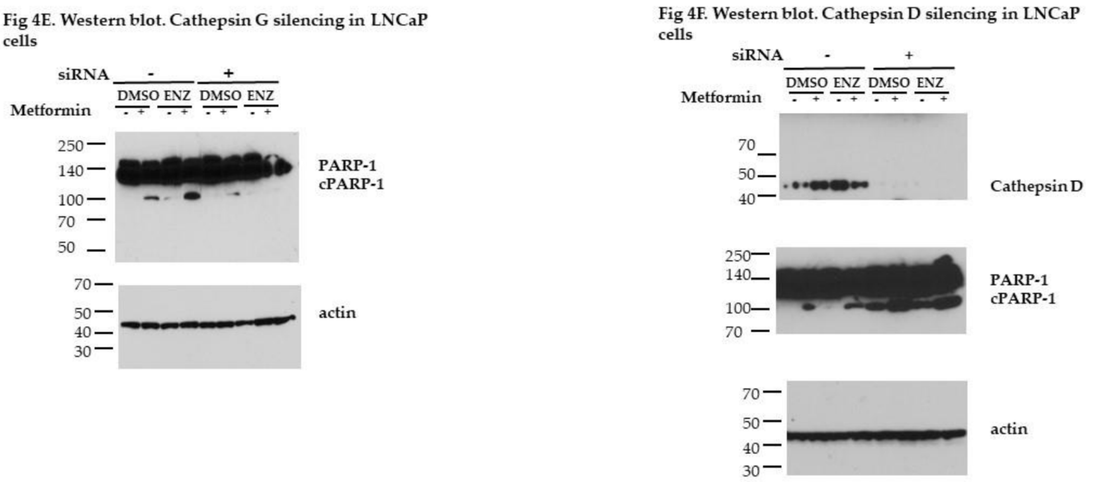

Figure S9. Original western blots for Figure 4C–F.

Original western blots for supplement Figure 5

S5

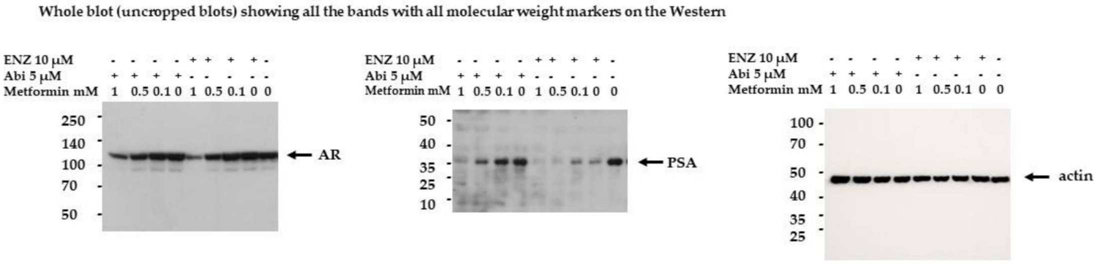

Figure S10. Original western blots for Figure S5.

Table S1. IC50 values of LNCaP cells.

Cell Line	Abiraterone (μM)	Enzalutamide(μM)	Metformin (mM)
LNCaP	7.5± 2.5	15 ± 2.5	3.5 ± 0.5
